# Partially observed bipartite network analysis to identify predictive connections in transcriptional regulatory networks

**DOI:** 10.1186/1752-0509-5-86

**Published:** 2011-05-27

**Authors:** Angel Alvarez, Peter J Woolf

**Affiliations:** 1 Department of Chemical Engineering, University of Michigan, Ann Arbor, MI 48109, USA; 2 Department of Biomedical Engineering, University of Michigan, Ann Arbor, MI 48109, USA; 3 Bioinformatics Program, University of Michigan, Ann Arbor, MI 48109, USA

## Abstract

**Background:**

Messenger RNA expression is regulated by a complex interplay of different regulatory proteins. Unfortunately, directly measuring the individual activity of these regulatory proteins is difficult, leaving us with only the resulting gene expression pattern as a marker for the underlying regulatory network or regulator-gene associations. Furthermore, traditional methods to predict these regulator-gene associations do not define the relative importance of each association, leading to a large number of connections in the global regulatory network that, although true, are not useful.

**Results:**

Here we present a Bayesian method that identifies which known transcriptional relationships in a regulatory network are consistent with a given body of static gene expression data by eliminating the non-relevant ones. The Partially Observed Bipartite Network (POBN) approach developed here is tested using *E. coli *expression data and a transcriptional regulatory network derived from RegulonDB. When the regulatory network for *E. coli *was integrated with 266 *E. coli *gene chip observations, POBN identified 93 out of 570 connections that were either inconsistent or not adequately supported by the expression data.

**Conclusion:**

POBN provides a systematic way to integrate known transcriptional networks with observed gene expression data to better identify which transcriptional pathways are likely responsible for the observed gene expression pattern.

## Background

Significant effort has been invested in identifying which genes regulate the expression of which other genes in a given genome[1-3]. The bioinformatics community has collected many of these gene-gene regulatory relationships into transcriptional networks that provide a global view of how gene regulation is orchestrated. For example, TRANSFAC collects protein-DNA binding interactions to identify potential gene regulatory mechanisms[4]. Similarly, RegulonDB provides a hand annotated regulatory network for the *E. coli *genome[5]. As more data become available, these transcriptional regulatory networks will become increasingly complete in the sense that they will describe the set of possible mechanisms for regulating each gene.

However, even with a fully complete and accurate transcriptional regulatory network, only some of the regulatory relationships will be relevant for a given cellular environment. For example, some gene regulatory mechanisms may only be used in rare cases of stress, or during a short developmental stage. In these cases, these rarely used regulatory mechanisms are correct, but largely non-predictive and as such may not be relevant to the process under study. In these specific cases, the general regulatory network is less useful.

**Figure 1:**
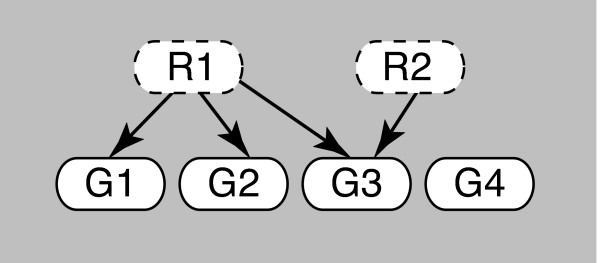
**A partially observed bipartite network**. A POBN model of a mechanism based on 2 unobserved regulators associated with 4 observed genes G1-G4.

In this paper, we introduce a Bayesian network based method to differentiate predictive and non-predictive connections in a transcriptional regulatory network given a body of gene expression data. The method we term Partially Observed Bipartite Network, or POBN, uses a simplified Bayesian network topology to describe a regulatory network, as is illustrated in Figure 1. A POBN has a top layer of unobserved regulators (protein activities) that connect to a lower level of observed variables (mRNA expression values). By casting the regulators as unobserved, a POBN makes it explicit that the activities of the regulatory proteins are unknown. As a first approximation, the activity of a regulator could be modeled as simply proportional to the mRNA expression level of a transcription factor, however this approximation ignores other regulatory events that are known to influence the regulatory process. For example, the activity of a regulator may be influenced by post-translational modifications, changes in protein localization, sequestration, and/or cleavage--all of which are mediated by other pathways in the cell. Unfortunately, this more complete view of transcriptional factor activity is complex, poorly understood, and difficult to quantitatively model. To circumvent this problem, the POBN approach allows the expression of the target genes to dictate the likely activities of each regulator. In doing so, POBN strives to identify regulatory topologies that are maximally consistent with both the expression data and the known regulatory network, while not specifying the mechanistic details that lead to the particular state of the regulatory proteins.

Expression data for learning these regulatory relationships can be divided into two classes: time series and static. Time series data consist of periodic measurements of a sample to obtain a time varying gene expression profile, while static data consist of a set of expression measurements made under different conditions, treatments, or sample types. If designed correctly, a time series study can identify the sequence of events that trigger a regulatory event, as has been widely explored elsewhere[6-8]. In contrast, a static study can only be used to infer relationships between regulators without a clear picture of the sequence. Methods to use these static data for transcriptional regulatory network analysis have been less widely explored, although a majority of the gene expression data collected are static. For example, over 80% of the expression data in the public repository GEO are from static measurements. Although more challenging, in this work we have chosen to explore how these static data can be used to infer regulatory networks using POBN.

To test the performance of the POBN algorithm for regulatory network reconstruction, we focus on the regulatory networks in *E. coli*. The regulatory network in *E. coli *is one of the best characterized, giving us a clear picture of the network we expect to find. In addition, digital resources such as the RegulonDB database provide the regulatory network in a machine readable form that is suitable for comparison to the POBN results.

## Methods

In the following sections we describe the algorithms and sample studies used to evaluate the POBN algorithm's ability to identify predictive and non-predictive regulatory relationships based on gene expression data.

### Gene expression data

We tested POBN using a set of 266 gene expression profiles in *E. coli *described elsewhere[9], and available online on GEO as GSE6836. This dataset represents a diverse range of biological backgrounds and environmental conditions including genetic perturbations, drug treatments, different growth phases, and a range of metabolic states. The study is well suited to this work because it contains many samples and covers a range of perturbations.

The selection of genes that could be meaningfully analyzed using POBN was based on the following two criteria: (1) genes must exhibit differential expression in at least some samples; and (2) genes must be present as target genes in the RegulonDB regulatory network. The first criterion was enforced by selecting the 300 genes with the largest variation as measured by the magnitude of the standard deviation of the expression value across samples. This selection approach will tend to favor genes with larger absolute expression levels, and a diverse range of expression values across the samples. When the second criterion was applied, only 189 of the 300 genes were found in the RegulonDB network as targets, producing a final list of 189 genes.

### Data discretization

For computational efficiency, the scoring metrics used in this study require that the data be discretized. Data were binned into three states, high, medium and low, with the top third of the values assigned to high, the bottom third assigned to low, and the remaining values assigned to the medium bin. This even sized binning strategy is widely used for discretization of gene expression data in the systems biology community[10-13], and has been shown empirically to be robust in capturing relevant details of the systems under study. We note that the POBN method can be used with continuous values directly, however the continuous scoring algorithms that are currently available are computationally impractical for networks involving more than a few hidden nodes. Instead, POBN scores the probability of each network using discrete data as described below.

### Scoring method

The regulatory bipartite networks tested here are modeled as a Bayesian network with the regulators as hidden nodes. In this network, variables are modeled using a multinomial model with Dirichlet priors, as is described elsewhere[14-16]. Using a multinomial likelihood and Dirichlet parameter priors leads the BD scoring metric that is used in this work[14,17]. This means that the parameter priors are modeled as Dirichlet distributions, and helps to reduce over-fitting. See the additional file 1: scoring_metric.doc for a more detailed discussion about Bayesian networks with hidden nodes. Below we provide a brief summary of the modified sampling-scoring method used in this work.

The end goal of our method is to test each single regulatory connection (regulator-gene associations) in a bipartite network to identify the connections that are more predictive than others based on a specific data set. For this, a score or a likelihood of the network with all the initial connections present needs to be evaluated. Then, a connection is removed and a new score for the resultant network without the connection is evaluated. The non-predictive associations can be identified as the ones that improve the initial network score once they are disconnected.

In most Bayesian network problems, a completely observed data set is used to estimate the likelihood or score of a network with the metric explained in the additional file 1: scoring_metric.doc. The scoring process with complete data is fast and thousands of networks can be score in minutes. However, in the networks studied in this work, the activity of the regulators is not observed which produce a data set with numerous missing entries. One way to handle these missing entries is to use Gibbs sampling, as is described in additional file 1: scoring_metric.doc and elsewhere[18-21]. However, using Gibbs sampling alone requires that all possible disconnections must be scored and sampled --a process that is computationally impractical when many missing entries are present.

To overcome this computational limitation, we have developed a new sampling-scoring approach to evaluate the score of a network each time a regulator-gene connection is eliminated. This new POBN approach is described below.

The sampling and scoring was done using PEBL, a python library previously developed in our group[17]. PEBL evaluates the probability of a discretized dataset given a topology using the BD (Bayesian Dirichlet) scoring metric described elsewhere[14]. The source code for PEBL is freely available online (http://code.google.com/p/pebl-project/).

The scoring process in our simulations comprised two main steps: (1) Gibbs sampling of the missing entries using the initial global graph and the discretized data and (2) calculating of an average score across the missing entries samples. These missing entries samples are set of different states configurations for the missing values. One set of configurations includes a complete round of states values (a single sample for each of the missing entries). The average score for each network was estimated using the BD metric and the sampled states for the hidden nodes obtained in step (1). This scoring process was carried out for the initial network and each single edge removal from that initial network. Note that Gibbs sampling was done only for the initial network. The same set of states configurations were used to estimate the average score of each single edge removal. This approximation assumed that the number of predictive edges is greater than the non-predictive ones, thus contributing significantly more to the network score.

We used an empirical approach to estimate the required number of saved configuration states after Gibbs sampling to estimate the score for any network. The criterion used to define a connection as predictive or non-predictive was the rank of the initial global network based on score relative to all the tested edges disconnections (score of networks after each disconnection). The connection evaluation process (predictive vs. non-predictive) will be explained in the next section. Based on this approach, set of configuration sates after Gibbs sampling with sizes of 100, 250, 500 and 1,000 rounds were tested for a first POBN optimization cycle. We observed insignificant changes between the results obtained for 250, 500, and 1,000 rounds of sampling. For these 3 set of configuration states sizes, 3 non-predictive edges out of 75-78 cast as non-predictive (as described in the next section) were observed to be different between each set results. Nevertheless, the networks evaluated after disconnecting these three discrepant edges ranked just below the criterion established to cast the edge as predictive/non-predictive. For the 100 rounds of states configurations case, the difference was of 6 edges. As a result of this analysis, all subsequent POBN optimization cycles were run by collecting 250 configuration states after Gibbs sampling of the initial network as a balance between accuracy and computational efficiency.

### Network searching and optimization process

The filtered 189 genes were mapped to target genes in the RegulonDB network. This mapped network produced a bipartite graph between regulators and targets containing 570 connections between 62 unobserved regulators and the selected 189 genes.

Given this network as an initial global network, we examined all one-edge removals in this network, resulting in 570 additional networks to be scored (one per edge removal), for a total of 571 networks. A complete list of these connections present in our initial global network is provided in the additional file 2: initial_global_net.xls.

**Figure 2:**
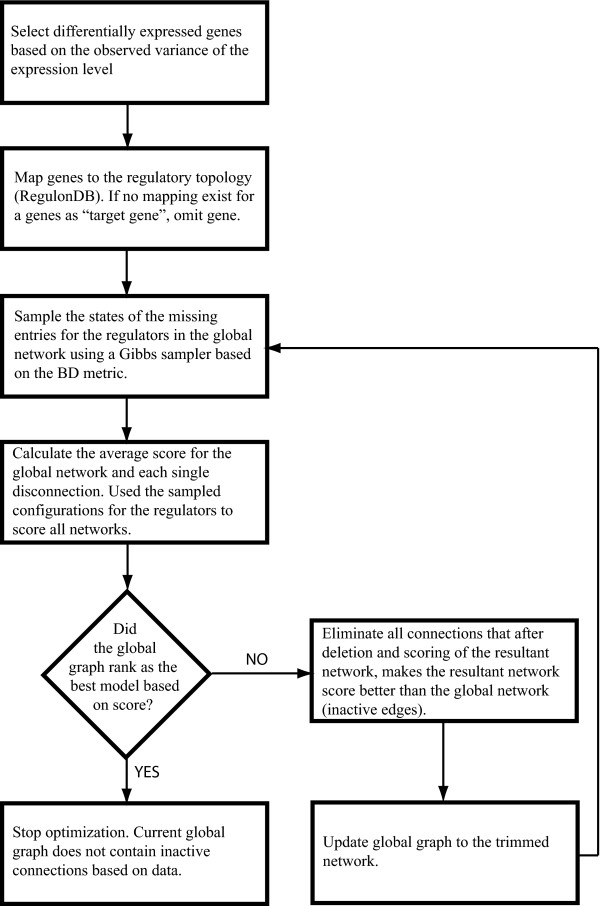
**POBN algorithm conceptual diagram**. Note that the creation of the non-predictive edges list is based on the rank of the network after an optimization round. If a connection is eliminated and the resulting model has a worse score than the initial network, this connection should stay as it plays an explanatory role based on the data. Otherwise, the connection is listed as non-predictive and eliminated from the global network.

If removing an edge in the global network improved the score then the disconnected edge was labeled as non-predictive. If the initial global network did not rank first on the score list, we proceeded with another scoring round (POBN cycle) after updating the initial global graph by eliminating the set of non-predictive connections. These steps were repeated until the starting global regulatory network (initial network for each cycle) ranked first on the list as the best network. Figure 2 shows a conceptual flow diagram of this process. Source code for the optimization is provided in the supplementary material additional file 3: POBN_single_round.py. The data and network format needed by PEBL is also explained in this same file.

### Synthetic Network Validation

To assess the POBN algorithm's discriminatory capability, we tested the algorithm using a synthetic dataset where the predictive and non-predictive edges are known beforehand. First, a bipartite network was defined with 4 regulators and 9 response variables. Each response variable was assigned to have 1-3 parents for a total of 11 connections in the synthetic network. After defining the topology, conditional probabilities were assigned for all nodes based on connectivity and the 3 discrete values that were allowed. Based on these parameters, 5,000 samples were generated to create a data set simulating a discrete static data set, full details of which are provided in the supplementary material. See additional file 4: synthetic_data_generation.py for the data generation script.

After sampling, the values of the 4 regulators were removed, making these regulator variables unobserved. Next, all combinations of possible non-predictive connections were added to the initial graph one at a time for a total of 25 cases, i.e. 25 = (4 regulators)*(9 response variables)-(11 defined connections). Once a single non-predictive edge was added, the weight for all edges in the graph were evaluated as described in the Scoring Method section above. This same process was repeated for all combinations of pairs of non-predictive edges that could be added to the original graph (300 pair addition cases).

## Results

### Synthetic data set

The synthetic case study provides a way to evaluate POBN's ability to discriminate between non-predictive and predictive edges with a known network. When a single non-predictive edge was added in all possible positions, 23-22-24 out of 25 non-predictive edges were correctly identified in three independent runs, and in no case was a true edge called non-predictive. For the cases where POBN did not call the added edge non-predictive, the non-predictive edge was ranked as the first or second weakest (score just below the initial graph). When a pair of non-predictive edges were added in all possible positions, in 282 out of 300 cases the non-predictive edges were correctly identified and in 13 of the remaining 18 cases the connections were ranked as the first or second weakest. Here again, in no case was a true edge called non-predictive. In runs with fewer samples the number of hits were lower but the algorithm was consistently precise in not calling a true edge non-predictive.

Overall, these synthetic results indicate that the POBN algorithm is frequently able to correctly identify the added non-predictive edge(s) with no observable false positive rate and a moderate false negative rate. This result suggests that the edges POBN identifies as non-predictive in the biological dataset will likely be non-predictive, however, edges deemed predictive may be only marginally so, depending on where they appear relative to the initial network.

### Non-predictive connections predicted using gene expression data

When the POBN analysis was applied to experimentally gathered gene expression data, the algorithm identified 93 non-predictive connections out of the 570 connections present in the initial regulatory network from RegulonDB. From this total, 75 non-predictive connections were found during the first sampling-score-ranking cycle of POBN analysis, 13 in a second cycle, and the last 5 in a third cycle. After the third optimization round, the score of the initial graph was no longer improved by removing any edges. During all the rounds of optimization, we observed that well characterized regulatory associations in *E. coli *such as the genes regulated by LexA (recA, recN, sulA, umuD, lexA) and AraC (araA, araB) were within the group of the best scoring edges in the network. A complete list of the 93 connections cast as non-predictive and the final trimmed network is provided in additional file 5: removed_edges.pdf and additional file 6: trimmed_net.xls. The additional file 7: initial_global_net.dot and the additional file 8: trimmed_graph.dot contain a graphical representation of the initial global network and the trimmed network.

## Discussion

By integrating a specific expression data set and a global regulatory network, POBN is able to identify a simplified regulatory network that is both mechanistically sound and maximally consistent with the expression data. This simplified network suggests which connections are of particular importance in the expression data.

During the optimization process, we observed that POBN tended to remove edges from genes that had the higher connectivity (>3 parents). In the initial regulatory network from RegulonDB, each target gene had between 1-9 parent regulators. After POBN optimization, the connectivity range was between 1-6 regulators per gene with most genes having between 1-4 parents.

**Figure 3:**
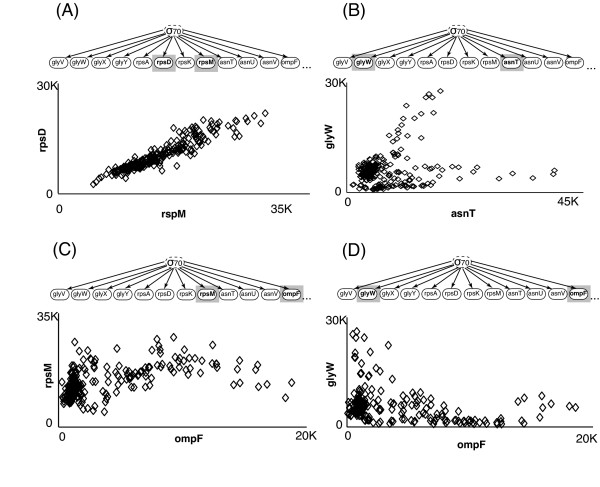
**Expression value comparisons between siblings**. The expression of all the genes shown are associated by the same sigma factor sigma70. Panels (A) and (B) compare the expression values of genes that originally appeared associated only by the same sigma factor in the global regulatory network. The genes rspD and rspM shown in (A), are nearly linear related, while glyW and asnT shown in (B) have a more complex relationship. After POBN optimization, ompF gene was disconnected from 8 of its 9 originally regulators in the global regulatory network. Panels (C) and (D) illustrate two examples of the association of ompF with genes under this same single sigma factor sigma70. Note that different but clear non-linear associations are observed between ompF and its siblings.

An extreme example of regulator trimming took place for the gene ompF. This gene is associated with 9 regulators based on the RegulonDB database. After optimization, 8 regulatory connections were removed from ompF suggesting that, based on the data set under study, the expression of this gene is better explained by 1 regulator rather than 9. Upon examination of the siblings of ompF in the optimized network (e.g. the genes that are also regulated by the same single parent), we see a regular, but nonlinear relationship between the gene expression values of ompF and its siblings (see Figure 3).

### Sigma factor sigma70

Note that even when the sigma factor sigma70 is considered a global factor needed for the transcription of most genes and its elimination from the initial network could simplify the computational analysis, we decided to keep it as part of the network to be optimized for the following two reasons: (1) The global network from RegulonDB contained several genes that had high variance in their expression data across samples and were only associated with sigma70. These genes were primarily associated with ribosomal subunits, t-RNAs, and ATP/metabolism processes. Because the purpose of POBN is to evaluate a general regulatory network knowledge in the context of a specific dataset, we kept sigma70 as it was the only known entity associated with the transcription of these highly expressed group of genes. (2) Transcriptional regulation often involves the co-action of different proteins and factors. One of the challenges when analyzing biological data is the presence of combinatoric phenomena that accurately explain the expression variances. If sigma70 participates in a combinatoric effect, eliminating sigma70 will reduce the accuracy of the network and the POBN analysis in general. As an example of this combinatoric effect, consider the regulation of genes yfiD, aceE and aceF suggested in RegulonDB. In the network from RegulonDB these genes appear regulated by 4 transcription factors but sigma70 is only associated with aceE and aceF and not with yfiD. In cases like this, the decision to eliminate predictive/non-predictive associations between regulators-genes could be affected if sigma70 is removed.

To evaluate the importance of sigma70 in the analysis, we also tested POBN with an initial network not including sigma70. The initial network was reduced from 570 regulatory associations to 402 (168 connections less corresponding to sigma70). After the simulation, POBN identified 32 non-predictive connections, all of which were predicted before as part of the group of 93 non-predictive associations when sigma70 was part of the initial network. The remaining 61 not included within these results consisted of 4 connections associated only with sigma70 (4 genes that were eliminated from the network as part of the new assumption) and 57 connections associated with other transcription factors. Most of these 57 connections, previously classified as non-predictive associations when sigma70 was part of the initial network, were associated with dual regulators such as FNR, CRP, ArcA, IHF and H-NS. In addition, this group of 57 connections ranked within the weakest 15% group of the total connections based on score suggesting that the initial not-relevant prediction for these connections were not far from reality. Sigma70 is biologically associated with the target genes of these dual regulators (FNR, CRP, ArcA, IHF and H-NS) and is part of the requirement for the transcriptional regulation to start. Nevertheless, global regulators and/or factors like sigma70 may require special considerations on computational analysis and is a topic of future work.

One may ask why any edges should be removed from the annotated regulatory network at all. Presumably the annotated network is well validated experimentally, and as such should represent our best prediction of the gene regulatory relationships. Based on the analysis here, we see two possible explanations. First, it is possible that the edge identified as non-predictive is not actually a regulatory relationship at all. In this case, the regulatory relationship present in RegulonDB would be an error or missannotation in the database. A second more likely reason for an edge to be called non-predictive is that the regulatory association is biologically correct, but is not predictive in the gene expression samples used in this study. In this latter case, removing the regulatory connection does not suggest that the there is no biological mechanism for the relationship, but instead that the connection is not relevant to the study. POBN cannot distinguish missannotations vs. non-predictive interactions, but either case represents unwanted pieces of information for the system under study. In both cases, POBN will favor reducing the number of regulators for each gene and consequently reducing the complexity of the model with little or no impact on the model's ability to predict the observed gene expression data even when the exact reason for the disconnection cannot be distinguished.

**Figure 4:**
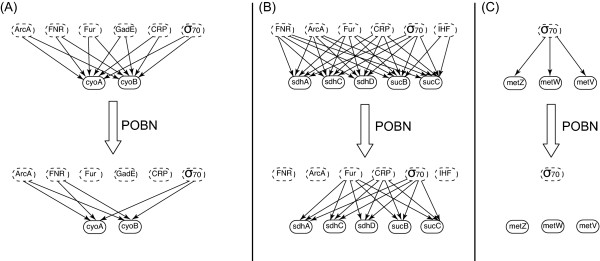
**Optimization of genes grouped in the same operon**. The networks in the top section are sub-networks of the original global network. Each sub-network has the original connectivity for the genes as suggested by RegulonDB and the target genes in each case form part of the same operon. In each case, each gene has an identical parent set. Note that in the three cases, the same non-predictive connections were predicted for each gene within each group.

It was consistently observed that cases of genes in the same operon were disconnected from the same regulator or group of regulators after POBN optimization. In other cases none of the gene members of an operon were disconnected at all. See Figure 4 for some examples. This was expected to happen because, in most cases, genes in the same operon are regulated by the same group of regulators. It is interesting to notice that even when POBN visited each gene individually with no operon definition during the simulation, it was consistent in disconnecting (or not) all the member genes in an operon from the same regulators. Note that there can be few cases of complex operons that can transcribe subsets of genes by means of different promoters and internal transcriptional units within the same operon[22]. For example, in Figure 4.b the operon grouping the shown genes consists of three transcriptional units with three different promoters. That is why RegulonDB has the regulator IHF associated with only sucB and sucC and not with the rest of the members of the operon. After POBN, these two genes sucB and sucC were disconnected from IHF and also from the same regulators that the other members of the operon were disconnected (FNR and ArcA). This suggests that all the genes in this operon were transcribed by mean of a common promoter.

The regulation of genes can be a dynamic and competitive process in which genes might need the co-action of other regulators and/or compete for regulatory binding sites. In addition, some regulators can have dual regulatory roles. This explains why in some cases a regulator or a combination of regulators were predicted to be the main control entities for the expression of some genes while in other cases the same regulators were disconnected from their target genes. An example of this can be observed in Figure 4. In Figure 4.a the expression of the genes are better explained by ArcA and FNR and POBN suggests that Fur, CRP and GadE does not contribute much. In contrast, for the genes illustrated in Figure 4b, the expression variance is predicted to be better explained by Fur and CRP and not by ArcA and FNR.

The genes metZ, metW, and metV illustrate an interesting ability of POBN, as all of these genes are associated with only sigma70 in the prior global network from RegulonDB. After optimization with POBN, these methionine t-RNA coding genes ended with no parents at the end of the analysis as shown in Figure 4C. A possible explanation for this parent elimination is that, for these genes, the prior network in RegulonDB did not have a complete list of possible regulators. There is evidence in the literature that under grow rate perturbations, the factor for inversion stimulation Fis, is been known to drastically alter the tRNAs pool composition including methionine tRNAs[23]. The intracellular concentration of this global regulator (Fis) varies substantially in response to changes in the nutritional environment and growth phases[24], conditions present as part of the samples set used in this study. It is possible that the expression variance for these genes metZ, metW and metV is better explained by a different regulator, i.e Fis instead of only sigma70. These results suggest that even when the true regulator is missing, POBN can still discriminate between consistent and inconsistent connections.

## Conclusion

By using a Bayesian network based approach the POBN algorithm is able to identify both linear and nonlinear relationships. For example, the regulatory relationship shown in Figure 3 includes both linear and nonlinear relationships. By identifying both kinds of relationships, POBN is able to detect relationships between genes that are not possible to detect using linear methods or most commonly used clustering methods.

In a larger context, the POBN approach provides a general way to integrate static observational data with knowledge about known regulatory relationships. In the example provided here, we found networks that were maximally consistent with both a set of gene expression data and a gene regulatory network. One could use a similar approach to identify relevant or predictive signaling pathways or protein phosphorylation networks from a mixture of experimental data and known topologies. By using POBN, all members of the network need not be directly observed, as long the measurements that are used in some way reflect the activity of the unobserved nodes.

## Abbreviations

**POBN**: Partially Observed Bayesian Network

## Authors' contributions

A.A. Designed the POBN approach and software, carried out the analysis, and wrote the paper

P.J.W. Designed the POBN approach, guided the analysis, and edited the paper

All authors read and approved the final manuscript.

## Supplementary Material

Additional file 1 **Static Bayesian networks and missing data**. This document provides more details about the Bayesian network metric used in this study as well as the Gibbs sampling approach used. Click here for file

Additional file 2 **Initial global network**. Table shows all the regulatory associations for the E. coli genes analyzed in this study: 189 genes with their 62 regulators as suggested by RegulonDB. Click here for file

Additional file 3 **POBN single round script**. This python script shows the steps to execute a single POBN optimization round given an initial network and gene expression data. Click here for file

Additional file 4 **Synthetic data generation script**. This python script shows the steps to generate a synthetic data set to test POBN. Click here for file

Additional file 5 **Edges cast as non-predictive**. This table shows all the non-predictive regulatory associations found in this study. Click here for file

Additional file 6 **Trimmed E. coli network**. Table shows all the regulatory associations for the E. coli genes after POBN optimization: 185 genes and 46 regulators Click here for file

Additional file 7 **Initial E. coli global network**. This file contain all the regulatory associations for the E. coli genes analyzed in this study in a "dot" graphic format: 189 genes and 62 regulators. Click here for file

Additional file 8 **Trimmed E. coli network**. This file contain all the regulatory associations for the E. coli genes after POBN optimizatuon in a "dot" graphic format: 185 genes and 46 regulators. Click here for file
