# Supplementary material for: Partially observed bipartite network analysis to identify predictive connections in transcriptional regulatory networks
Source: BMC Syst Biol. 2011 May 27;5:86. doi: 10.1186/1752-0509-5-86 (PMC3117734; doi:10.1186/1752-0509-5-86)
Supplement: Additional file 1 — Static Bayesian networks and missing data. This document provides more details about the Bayesian network metric used in this study as well as the Gibbs sampling approach used. [file 1752-0509-5-86-S1.DOC]

**Static Bayesian networks and missing data**

Here we describe the scoring metric and the Gibbs sampling method used in this work. Additional theoretical details for these numerical and computational tools can be found in the referenced documents provided in the bibliography.

*Background: Static Bayesian networks*

Bayesian networks can be used to describe causal or apparently causal relationships in data. The algorithm for scoring the likelihood of a Bayesian network given data is based on a widely used and theoretically sound principle of probability theory called Bayes’ Rule. Bayes’ rule in this context is used to evaluate the probability that a model is true given a body of experimental data. Mathematically Bayes’ rule can be expressed as:

For a Bayesian network, a model is a directed acyclic graph. Nodes in this graph represent variables. Arrows between nodes represent probabilistic dependencies indicating a causal relationship between the two variables. These probabilistic dependencies can be estimated using experimental data or known facts that interrelate the variables. Hence, for each node, there is a conditional probability set of values that quantitatively describes the relationship between the node and its descriptors (parents).

Note that the graphical representation of a Bayesian network is similar to that of a kinetic model such as a signaling pathway, but is interpreted differently. In a kinetic model, edges represent a specific function (activation, repression, a linear relationship, etc.), or a transformation (e.g. A🡪B implies A becomes B). In a Bayesian network, these causal relationships may be any activation effect as well as inhibition and also includes linear, nonlinear, and/or multimodal associations between variables.

The term P(Model | Data) represents the probability that the model is correct given the observed data. P(Data) is not calculated as it is a constant in our expression, thus we will only compare relative scores. In the POBN analysis, P(Model) was either 1 or 0, for networks that were and were not allowed respectively.

The P(Data | Model) is the probability of the particular data configuration given the model. This term is calculated by marginalizing over all parameters in a specific model (conditional probability values associated with each node). In this work, connections between a gene and its regulator(s) are modeled as a discrete multinomial distribution with Dirichlet priors. By using a multinomial model, the network can capture both linear and nonlinear relationships. In addition, for a multinomial model, the term P(Data|Model) has a closed form solution described elsewehre [13-16]. This solution is known as the Bayesian Dirichlet metric (BD) and has the following form:

(1)

Where “*n”* is the total number of variables, “*qi*” is the total possible state configurations for a parent, “*ri*” is the number of states of a variable (arity), “*Nij*” is the number of cases parent of variable “*i”* is in state (or state combination) “*j*”, “*Nijk*” is the number of cases variable “*i*” is in state “*k*” and parent(s) in state “*j*”. The expression in Eqn. 1 describes the product of the probability of a variable being in a state *k* and the parents of this variable in a state *j*. The more informative the parents are of their child, the higher the value of P(Data | Model).

With the ability to score a network in hand, computer software packages have been developed to score networks based on a given data set. Furthermore, complex topological learning tasks have been included by using a wide range of tools from the field of discrete optimization including Monte Carlo methods, greedy learning, simulated annealing, and genetic algorithms to name a few . For a comprehensive list of Bayesian network software packages, see the online list by Kevin Murphy. In our work, we used PEBL a python library previously developed in our group, to estimate the score of a network give a dataset.

Because we are modeling our regulatory models as a bipartite network, network learning and scoring for POBN is simpler than the general Bayesian Network learning problem. Below we describe how to handle the missing values of the regulatory proteins when scoring a network.

*Estimating a BDe metric value (network score) with missing data*

A key challenge in identifying regulatory relationships is the lack of data on the activity of the regulators themselves. With these values missing, we used a modified method to estimate the score of a network where the activity of the regulator is assumed to be unknown. A simple but computationally unfeasible way to evaluate the score of a network with missing values is to marginalize over all possible state configurations for the missing entries and then take an average. However, the number of all possible state configurations increases exponentially with the number of missing values, making this exact marginalization impractical. For example, in a small system with 2 missing binary variables and 10 observations there are more than a million possible different state configurations.

An alternative to exact enumeration is to selectively sample the configurations space. To do this sampling, we used an MCMC method known as Gibbs sampling. Gibbs sampling is commonly used in computational statistics, and has found extensive use in Bayesian networks score estimation with missing entries. In general, Gibbs sampling works in the following way:

• Values for all unobserved entries are randomly chosen each time that a BD metric score needs to be estimated.

• A randomly chosen unobserved entry is re-sampled based on the probability of each of the states for the visited variable as calculated with P(Model | Data):

- The score of the network is estimated for each of the possible states that the variable can assume, keeping the rest of the random values assigned to the other variables intact.
- From the normalized scores evaluated for each of the possible states, take a random sample from the scores distribution and keep that value for the variable until the variable is visited again.

• The new sampled value for an entry is used when evaluating a future entry for another variable.

The last two steps are repeated many times. When each variable has been visited once, we say that a first “round of sampling” is complete. For each individual rounds, a complete sub-data set is generated with a corresponding score from it. Many rounds of sampling are kept to estimate an average score at the end. It is a common practice to discard the first rounds of samples (burn-in period) and consider only rounds after the n^th^ eliminated rounds. Note that Gibbs sampler does not select a single best data configuration (a single round with specific values for the hidden entries), but instead samples a wide variety of possible configurations for the hidden values, favoring the more likely configurations over the less likely ones.

The result of this calculation is an average probability score of the model given the available data.
