# Supplementary material for: Partially observed bipartite network analysis to identify predictive connections in transcriptional regulatory networks
Source: BMC Syst Biol. 2011 May 27;5:86. doi: 10.1186/1752-0509-5-86 (PMC3117734; doi:10.1186/1752-0509-5-86)
Supplement: Additional file 5 — Edges cast as non-predictive. This table shows all the non-predictive regulatory associations found in this study. [file 1752-0509-5-86-S5.PDF]

List of connections removed after POBN optimization. Regulators in first column were initially connected with the corresponding genes in the second column.

Regulator(s)	Gene(s)
CpxR, EnvY, Lrp, OmpR	ompC, ompF
FruR, IclR	aceB
GutM, GutR	srlA
Rob, SoxS	sodA
GalR, GalS	mglB, galE, galT
Fis	nuoB, nuoE, hupA
CRP	cyoA, cyoB, ompF
FNR	sucB, sucC, sdhA, sdhD, sdhC
ArgP	nrdB, nrdA
ArgR	nusA, metY
HU	galE, galT
ArcA	mdh, nuoB, sucB, sucC, aceB, sdhA, sdhD, treB, sdhC
CspA	hns
NarL	nuoB, nuoE
RstA	ompF
TorR	hdeB
Sigma70	serV, metW, metZ, metV
Fur	sodB, cyoA, cyoB, ompF, sodA
FlhDC	mglB, mdh
DgsA	ptsH
Sigma38	mglB, galE, galT, hdeB, hdeA
CdaR	rnpB
Sigma32	gapA
MarA	sodA, hdeB, hdeA
H-NS	sodB, srlA, galE, galT, cydB
GadE	cyoA, cyoB, hdeB
GadX	hns, hdeB
TreR	treB
IHF	sucB, sucC, nuoB, sodB, ompC, nuoE, aceB, ompF, sodA
